# ECRG4 acts as a tumor suppressor gene frequently hypermethylated in human breast cancer

**DOI:** 10.1042/BSR20190087

**Published:** 2019-05-10

**Authors:** Gao-Yan Tang, Guo-Jun Tang, Lu Yin, Chen Chao, Ren Zhou, Guo-Ping Ren, Jia-Yu Chen, Wei Zhang

**Affiliations:** 1 Department of Pathology and Pathophysiology, Key Laboratory of Disease Proteomics of Zhejiang Province, School of medicine, Zhejiang University, Hangzhou, Zhejiang Province, China; 2 Department of Surgical Oncology, Zhejiang Jinhua Guangfu Tumor Hospital, Jinhua, Zhejiang 321000, China; 3 Department of Pathology, Zhejiang Provincial Hospital of Traditional Chinese Medicine, Hangzhou, Zhejiang 310053, China; 4 Department of Pathology, The First Affiliated Hospital of Zhejiang University, Hangzhou 310003, China; 5 Department of Basic Medical Sciences, Shaoxing University Medical School, Shaoxing 312000, China

**Keywords:** Breast Cancer, DNA methylation, ECRG4

## Abstract

Human breast cancer is a malignant form of tumor with a relatively high mortality rate. Although esophageal cancer-related gene 4 (ECRG4) is thought to be a possible potent tumor suppressor gene that acts to suppress breast cancer, its precise role in this disease is not understood. Herein, we assess the correlation between ECRG4 expression and DNA methylation, probing the potential epigenetic regulation of ECRG4 in breast cancer. We analyzed ECRG4 promoter methylation via methylation-specific PCR (MSPCR), bisulfite sequencing, and a promoter reporter assay in human breast cancer cell lines and samples. Gene expression was assessed by quantitative real-time PCR (qPCR), while protein levels were assessed by Western blotting. CCK8 assays were used to quantify cell growth; Esophageal cancer-related gene 4 wound healing assays were used to assess cellular migration, while flow cytometry was used to assess apoptosis and cell cycle progression. Apoptosome formation was validated via CO-IP and Western blotting. We found that human breast cancer samples exhibited increased methylation of the ECRG4 promoter and decreased ECRG4 expression. Remarkably, the down-regulation of ECRG4 was highly associated with promoter methylation, and its expression could be re-activated via 5-aza-2′-deoxycytidine treatment to induce demethylation. ECRG4 overexpression impaired breast cancer cell proliferation and migration, and led to G0/G1 cell cycle phase arrest. Moreover, ECRG4 induced the formation of the Cytc/Apaf-1/caspase-9 apoptosome and promoted breast cancer cell apoptosis. ECRG4 is silenced in human breast cancer cells and cell lines, likely owing to promoter hypermethylation. ECRG4 may act as a tumor suppressor, inhibiting proliferation and migration, inducing G0/G1 phase arrest and apoptosis via the mitochondrial apoptotic pathway.

## Introduction

Breast cancer is a malignant form of tumor originating from breast epithelial tissue. Breast cancer occurs most frequently in women and has become one of the major diseases that threaten the health of women. It is a heterogeneous disease, with multiple risk factors including environmental, dietary, genetic, and epigenetic influences. Both oncogene activation and/or tumor suppressor gene (TSG) inactivation have been found to contribute to the occurrence and progress of breast cancer. Gene point mutations and deletions are the main causes of TSG inactivation in breast cancer. However, recent studies have revealed that epigenetic alterations, including aberrant promoter methylation and histone modification, may provide a novel mechanism for TSG silencing without the need for changes in nucleotide sequence. Several instances of promoter CpG methylation-based TSG silencing in breast cancer have been identified. For example, BRCA1, a classic TSG which plays an essential role in the occurrence and development of breast cancer, has been found to frequently exhibit promoter hypermethylation in breast cancer tissues [1,2]. Therefore, aberrant TSG methylation is linked with breast cancer pathogenesis, and is an important tumor marker in the context of this disease.

Esophageal cancer-related gene 4 (ECRG4) was first identified in normal human esophageal epithelial tissue, and performs pivotal functions with respect to cell proliferation, differentiation, migration, invasion, and death [3]. ECRG4 is also believed to be a TSG, as normal tissue levels of ECRG4 are high; whereas, it is down-regulated or silent in several types of tumor such as human esophageal squamous cell carcinoma, nasopharyngeal carcinoma, renal cell cancer, colorectal carcinoma, gastric cancer, and hepatocellular carcinoma [4–10]. Overexpression of ECRG4 was able to inhibit the proliferation, migration, and invasion of several tumor cells. Although recent studies have strongly suggested that ECRG4 is a potential potent TSG that suppresses breast cancer development, its precise role in this disease remains to be fully explored. To that end, we assessed the link between ECRG4 expression and DNA methylation in cell lines as well as in breast cancer samples, thus probing the potential epigenetic regulation of ECRG4. We observed that human breast cancer samples showed reduced ECRG4 expression, and this was associated with hypermethylation of the ECRG4 promoter. In the MCF-7 and BT483 breast cancer cell lines we also found that ECRG4 inhibits cell growth and migration, and that ECRG4 overexpression induces apoptosis, possibly via mitochondrial apoptotic pathway activation.

## Materials and methods

### Patient samples, cells, and regents

A total of 17 tissue samples of breast tumors and neighboring normal tissues were collected from the First Affiliated Hospital of Zhejiang University between 2013 and 2014. Human MCF-7 and BT483 breast cancer cell lines came from the Shanghai Cell Institute Country Cell Bank and grew in complete DMEM (Life Technologies) containing 10% fetal bovine serum (Gibco, Australia). Trichostatin A (TSA) as well as 5-Aza-2′-deoxycytidine (5-Aza-CdR) was obtained from Gene Operation and Sigma, respectively.

### Quantitative real-time PCR (qPCR)

RNAsio Plus (Takara) was used for extraction of total cellular RNA, of which 1 µg was utilized for cDNA reverse transcription via the PrimeScript RT Reagent Kit (Takara). The ORF of ECRG4 was amplified using specific primers.

SYBR Green-based quantitative real-time PCR (qPCR) (Takara) was conducted on an Exicycler™ 96 quantitative fluorescence analyzer (Bio-Rad) using specific primers, with the 2^−∆∆C^_t_ method used to quantify relative gene expression.

### Western blot

A lysis buffer containing PMSF (Beyotime) at 4°C was used to collect total protein. Cell lysates were denatured by boiling, separated on 12% SDS–PAGE gels, and transferred to PVDF membranes (Millipore). Membranes were blocked with 5% BSA in TBS/Tween and probed with appropriate primary antibodies after which an HRP-conjugated secondary antibody was used. Protein was visualized using an enhanced chemiluminescence (ECL) approach.

### Methylation analysis

A DNA Tissue Kit (OMEGA) was used to isolate total genomic DNA, of which 2 µg was used for bisulfite modification via the EZ DNA Methylation kit (Zymo Research). The primers for bisulfate sequencing were: upstream 5′- AGTGGGGGAGTTAAGGAGATA-3′, downstream 5′-CTCACCTAAACCCCAACACA -3′. The length of the amplified sequence was 545 bp, containing 49 CpG sites. The PCR amplifications were cloned into the PMD19-T vector, transfected into the trans5α cells, and then ten randomly selected colonies were sequenced.

For methylation-specific PCR (MSPCR), genomic DNA was treated with bisulfate, and subjected to PCR using sets of primers specific for unmethylated (U) or the methylated (M) forms of this sequence. These primers were as follows: M-forward primer: 5′-AGAGGATTTCGGTGGTATTCGTTC-3′; M-reverse primer: 5′-GACCGC GAATTATCCCTACG-3′. U-forward primer: 5′-GAGAGAGGATTTTGGTGGTAT TTGTTTG-3′; M-reverse primer: 5′-AACAAACAAACACAACCACAAATTATCCCT ACA-3′.

### Promoter reporter assay

Exponentially growing MCF-7 cells received 10 µM 5-Aza-CdR treatment for 2 days, and were subsequently co-treated with 1 µM TSA. The ECRG4 promoter region then underwent PCR amplification and was cloned into the pGL3-Basic vector (Promega, WI, U.S.A.). The recombination plasmid and the internal control vector pRL-HTK (Promega, WI, U.S.A.) were then co-transfected into MCF-7 cells using Lipotamine 3000 as a transfection reagent (Invitrogen). After 48 h, luciferase activity was assessed via Dual-Luciferase Reporter Assay System (Promega, WI, U.S.A.).

### Construction of cell lines stably overexpressing ECRG4

MCF-7 and BT483 cells grown to 70–80% confluence were infected with an ECRG4 lentivirus in the presence of Polybrene (8 µg/ml). After 48 h, we determined lentiviral infection efficiency based on green fluorescent protein (GFP) expression. The stably transfected cells were selected and enriched using puromycin (2 µg/ml). After 1 week, the infection efficiency (percentage GFP+) was again detected by flow cytometry.

### Cell proliferation assay

ECRG4-positive and ECRG4-negative MCF-7 and BT483 were plated in 96-well plates (5 × 10^3^ cells). After 1, 2, 3, and 4 day, cell viability was determined via CCK-8 assay.

### Flow cytometric analyses of apoptosis and cell cycle progression

For AnnexinV-PE staining, cells double stained using 5 µl AnnexinV-PE and 10 µl 7-AAD, and then underwent flow cytometric analysis. To analyze cell cycle progression, the harvested cells were PBS washed, then resuspended in 500 µl of appropriate staining solution, and subjected to flow cytometry analysis.

### Wound-healing assay

Layers of MCF-7 and BT483 cells were scratched using a sterile 10 µl pipette tip; thereby, generating a wound. Cells were then PBS washed to remove debris, and grown in serum-free DMEM. At the indicated time points (0 and 48 h), wound images were recorded to analyze cell migration.

### Co-immunoprecipitation

Cells were harvested and lysed using RIPA buffer, and then 2 µl anti-Aparf antibody (Abcam) was added to these lysates at 4°C for 6 h. Next, we added 10 µl protein A/G Plus-Agarose to this mixture for 12 h, and agarose beads were then collected, boiled in SDS sample buffer, and assessed via Western blotting.

### Statistics

Data was analyzed using Student’s *t* tests and ANOVA as appropriate, using SPSS v19.0. Data are given as means ± standard deviation. *P*<0.05 was the threshold of statistical significance.

## Results

### ECRG4 mRNA expression was down-regulated in breast cancer

**Figure 1:**
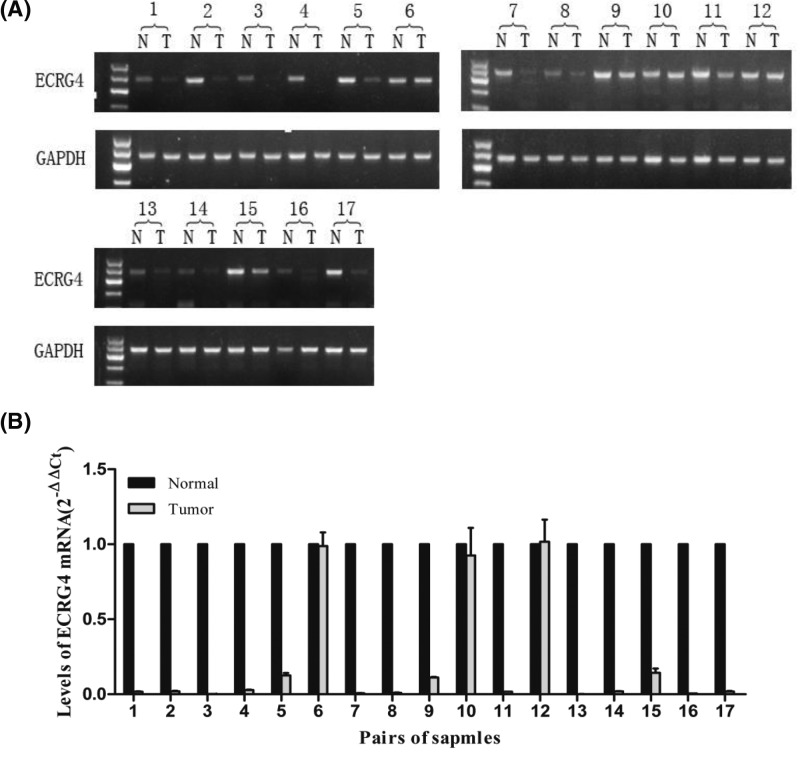
The level of ECRG4 mRNA in breast cancer tissues (**A**) Breast cancer and control non-tumor breast tissues RNA was subjected to RT-PCR (**B**) Breast cancer and control non-tumor breast tissues RNA was subjected to qPCR.

We first quantified the mRNA expression level of endogenous ECRG4 by qPCR assay in 17 donor tissues, comparing expression in pairs of breast cancer tissues and matched non-tumor tissues. We observed lower ECRG4 mRNA expression in 82.4% of tumor tissues (14/17) (Figure 1A,B). This result suggested that ECRG4 levels in breast cancer were frequently down-regulated (*P*<0.01).

### Breast cancer ECRG4 promoter methylation status

**Figure 2:**
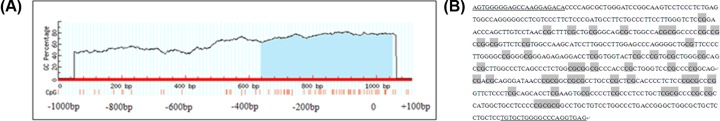
ECRG4 promoter status (**A**) A schematic structure of ECRG4 CpG islands. (**B**) The sequence of the hypermethylated region of the ECRG4 promoter. CpG sites are shaded.

We analyzed the ECRG4 promoter region between 1000 bp upstream and 100 bp downstream (−1000 to + 100 bp) of first exon using the MethPrimer software, and found the majority of CpG islands to be concentrated in the −400 to −100 bp region (Figure 2A). In total we found that the ECRG4 promoter region contains 49 CpG sites (Figure 2B). We next analyzed these 49 CpG sites, and found that the methylation frequency of these sites in tumor samples (82.35%) was much higher than in paired non-tumor tissues (Supplementary Figure 1).

### Down-regulation of ECRG4 is closely associated with promoter methylation

**Figure 3:**
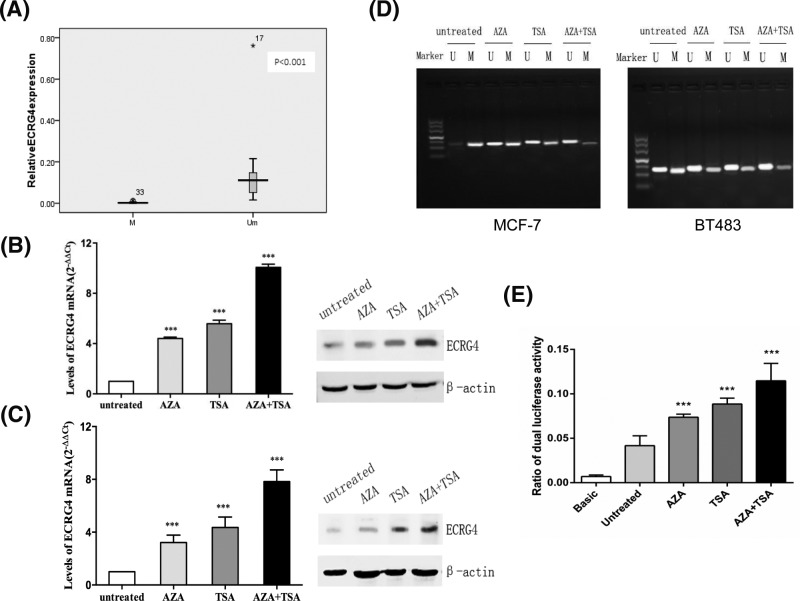
Down-regulation of ECRG4 was closely associated with promoter methylation (**A**) The correlation between ECRG4 promoter methylation and its mRNA expression level in breast cancer tissues (M: Methylated; Um: Unmethylated). (**B**) The effect of 5-Aza-CdR and/or TSA treatment on ECRG4 expression in MCF-7 cells as assessed via qPCR and Western blot. (**C**) 5-Aza-CdR and/or TSA treatment effects on ECRG4 expression in BT483 cells as assessed via qPCR and Western blot. (**D**) 5-Aza-CdR and/or TSA treatment effects on ECRG4 demethylation in MCF-7 and BT483 cells as measured by MSPCR. (**E**) T5-Aza-CdR and/or TSA treatment effects on ECRG4 promoter activity in MCF-7 cells as measured by luciferase reporter assay. ***P<0.001.

We next sought to determine whether down-regulation of ECRG4 was closely associated with the methylation of its promoter region. We found a negative correlation between ECRG4 promoter methylation and its mRNA expression (*r*=-0.634, *P*<0.001, Figure 3A). We therefore next assessed whether histone deacetylation and DNA demethylation influence the observed down-regulation in ECRG4 levels. MCF-7 and BT483 cells were treated with the DNA methyltransferase inhibitor 5-Aza-CdR and/or the histone deacetylase inhibitor TSA, and ECRG4 expression levels in MCF-7 and BT483 cells was measured via qPCR and Western blotting. We found that ECRG4 mRNA and protein expression in MCF-7 and BT483 cells was increased by 5-Aza-CdR treatment, and was further enhanced by TSA co-treatment (Figure 3B,C). These results suggested that 5-Aza-CdR and TSA co-treatment may synergistically facilitate the up-regulation of ECRG4 expression.

Next, the methylation status of ECRG4 was assessed by MSPCR assay. We found that ECRG4 promoter methylation was decreased following 5-Aza-CdR and/or TSA treatment. Most importantly, the combination of these agents appeared to exhibit a synergistic effect on the inhibition of ECRG4 promoter methylation (Figure 3D). We then conducted a luciferase reporter assay to analyze MCF-7 ECRG4 promoter activity. We found that the proximal region of the ECRG4 promoter (−400 to −100 bp) exhibited much high promoter activity upon 5-Aza-CdR and/or TSA treatment (Figure 3E). This suggests that hypermethylation of the promoter region of ECRG4 may be an important mechanism mediating the down-regulation of ECRG4.

### ECRG4 overexpression inhibits breast cancer proliferation and migration

**Figure 4:**
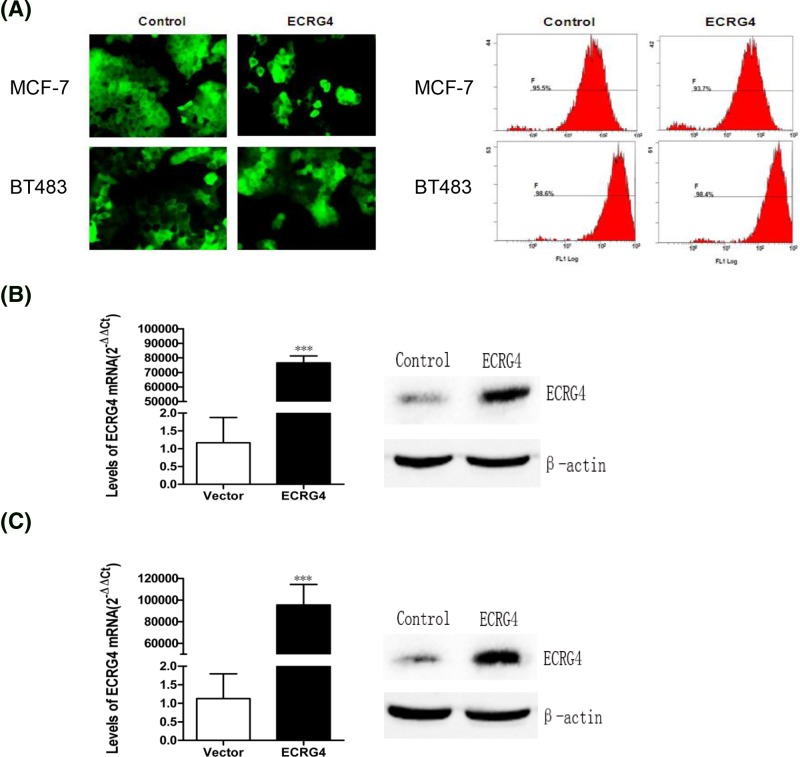
Construction and validation of stable cell lines overexpressing ECRG4 (**A**) Construction of stable cell lines overexpressing ECRG4. GFP expression was determined by fluorescence microscopy (×40) and flow cytometry. (**B**) Validation of the overexpression of exogenous ECRG4 in MCF-7 cells as assessed via qPCR and Western blot. (**C**) Validation of the overexpression of exogenous ECRG4 in BT483 cells as assessed via qPCR and Western blot. ***P<0.001.

**Figure 5:**
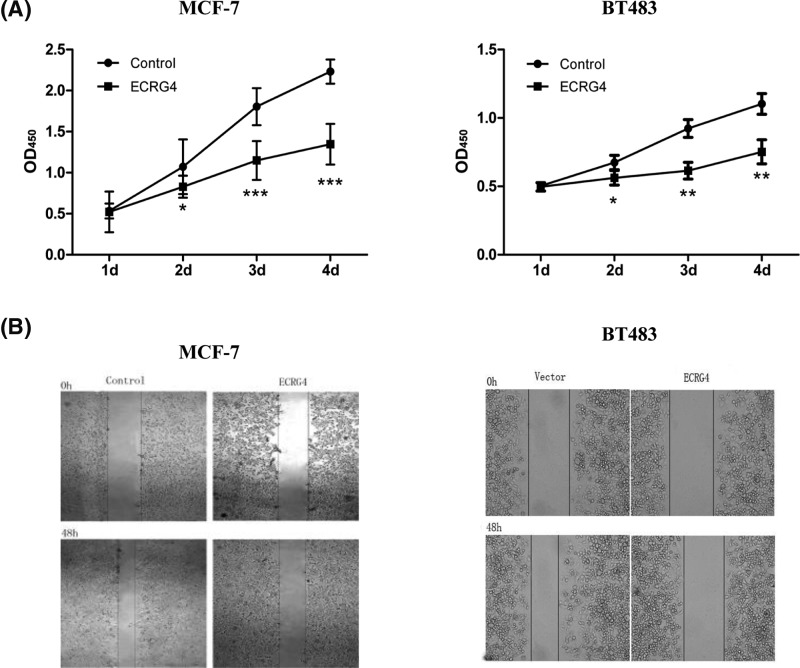
ECRG4 affects cell proliferation and migration in breast cancer cells (**A**) Effects on cell proliferation in MCF-7 and BT483 cells overexpressing ECRG4. (**B**) Effects on cell migration in MCF-7 and BT483 cells overexpressing ECRG4. *P<0.05, **P<0.01, ***P<0.001.

We next introduced exogenous ECRG4 into MCF-7 and BT483 cells via lentiviral transduction in order to generate stable ECRG4-overexpressing cells lines (Figure 4A). Using qPCR and Western blot, we confirmed that protein and mRNA levels of ECRG4 were both increased in ECRG4-overexpressing MCF-7 and BT483 cells (Figure 4B). We then investigated how ECRG4 overexpression affects cell viability, finding that it significantly inhibited cell proliferation in MCF-7 and BT483 cells (Figure 5A). Furthermore, we found that ECRG4 inhibited the migration of MCF-7 and BT483 cells (Figure 5B). This suggests that ECRG4 may inhibit breast cancer proliferation and migration.

### ECRG4 overexpression induces apoptosis via the mitochondrial apoptotic pathway

**Figure 6:**
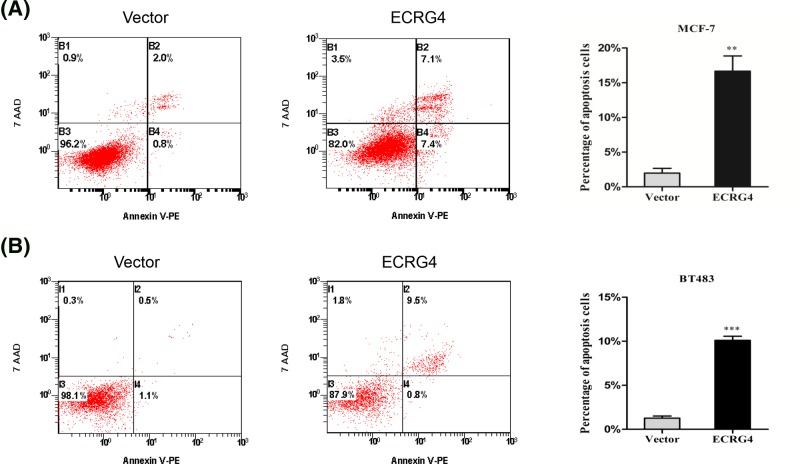
Overexpression of ECRG4 induces apoptosis in breast cancer cells (**A**) The effect of ECRG4 on apoptosis in MCF-7 cells as measured by flow cytometry. (**B**) The effect of ECRG4 on apoptosis in BT483 cells as measured by flow cytometry. **P<0.01, ***P<0.001.

**Figure 7:**
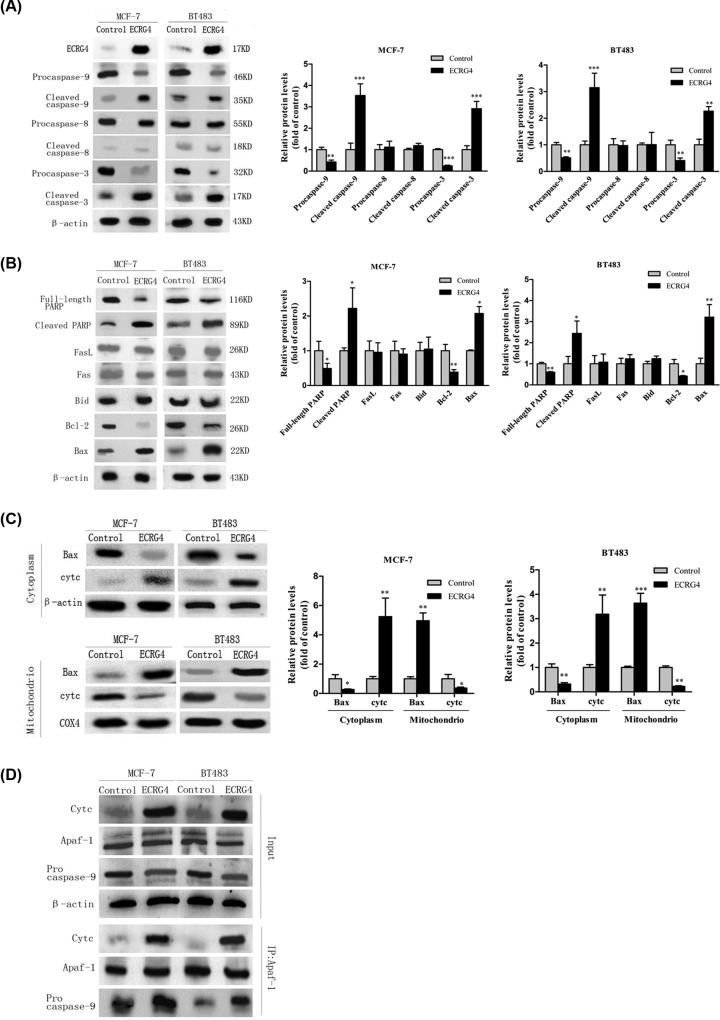
Overexpression of ECRG4 induces apoptosis in breast cancer cells (**A**) The effect of ECRG4 on caspase-9 and caspase-3 activation in MCF-7 and BT483 cells as measured by Western blotting. (**B**) The effect of ECRG4 on the protein level of Fas, FasL, PARP, cleaved-PARP, Bcl-2, Bid, and Bax as measured by Western blotting. (**C**) The effect of ECRG4 on Bax translocation and Cytc release as measured by Western blotting. β-actin and COX4 served as internal loading controls for the cytosolic fraction and the mitochondrial fractions, respectively. (**D**) The effect of ECRG4 on apoptosome formation. Cells were collected and immunoprecipitated using anti-Apaf-1, and caspase-9 and Cytc levels were then analyzed by Western blotting. *P<0.05, **P<0.01, ***P<0.001.

Given the reduction in cell viability, we next assessed how ECRG4 overexpression influences breast cancer apoptosis. We found that this overexpression induced MCF-7 and BT483 cell apoptosis (Figure 6). We then probed the possible mechanisms underlying this ECRG4-induced apoptosis. Caspases are known to play pivotal roles in apoptosis, with caspase-8 and caspase-9 serving as initiators of this process and caspase-3 serving as an effector, all of which are essential for mediating apoptosis. We found that overexpression of ECRG4 decreased levels of pro-caspases 3 and 9, while simultaneously increasing cleaved levels of these caspases (Figure 7A). In contrast, levels of both the pro and cleaved forms of caspase-8 were unchanged (Figure 7A). PARP is a cleavage substrate of activated caspase-3, such that the cleaved form of PRRP is an indicator of caspase-3 activation and is an important apoptotic marker. We found that ECRG4 induced clear PARP cleavage, leading to a decrease in levels of full-length PARP (116 kDa) and an increase in levels of cleaved PARP fragment (85 kDa) (Figure 7B). These results implied that ECRG4 may induce apoptosis via activation of caspases 3 and 9.

We next thought to determine the role of the death receptor pathway and the mitochondrial pathway in ECRG4-induced apoptosis. We found that ECRG4 promoted Bax expression; whereas, it reduced Bcl-2 expression. However, we did not detect clear alterations in Bid expression, consistent with the negative caspase-8 results observed. Together these results suggested that the mitochondrial pathway may be an important mediator of apoptosis induced by ECRG4 (Figure 7B).

Next, the location of Bax and Cytc in breast cancer cells was analyzed, leading us to find that levels of Cytc were decreased in mitochondrial fraction and increased in the cytosolic fraction, suggesting that Cytc may translocate from mitochondria to the cytosol. In contrast, we observed that Bax translocated from the cytosol to mitochondria, resulting in increased mitochondrial levels as well as a corresponding decrease in cytosolic levels (Figure 7C). We subsequently decided to investigate whether ECRG4 can induce the formation of the Cytc/Apaf-1/caspase-9 apoptosome by Co-IP and Western blot analysis. We found that anti-Apaf-1-precipited Cytc and caspase-9 was both increased in ECRG4-overexpressing cells (Figure 7D). Co-IP results further supported the conclusion that ECRG4 may promote apoptosome formation. Collectively, the above data indicated that ECRG4 may induce cell apoptosis primarily through the mitochondrial pathway.

### ECRG4 arrested breast cancer cells at G0/G1 phase

**Figure 8:**
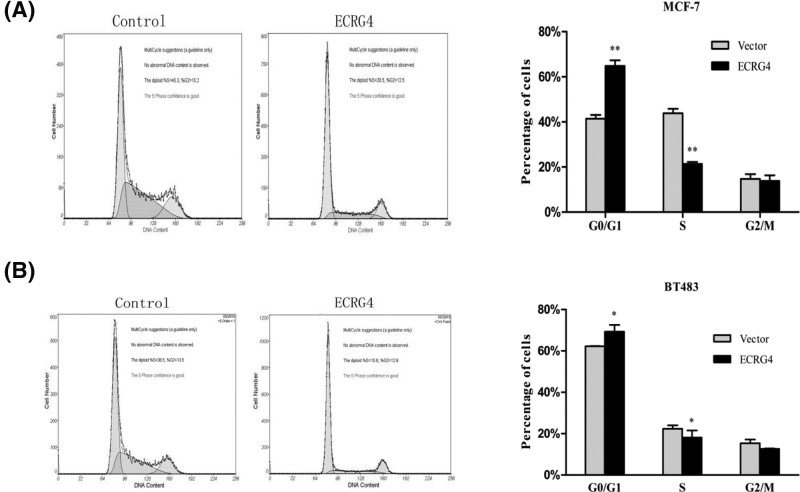
The effect of ECRG4 on the cell cycle in breast cancer cells (**A**) The effect of ECRG4 on the cell cycle in MCF-7 cells as measured by flow cytometry. (**B**) The effect of ECRG4 on the cell cycle of BT483 cells. *P<0.05, **P<0.01.

**Figure 9:**
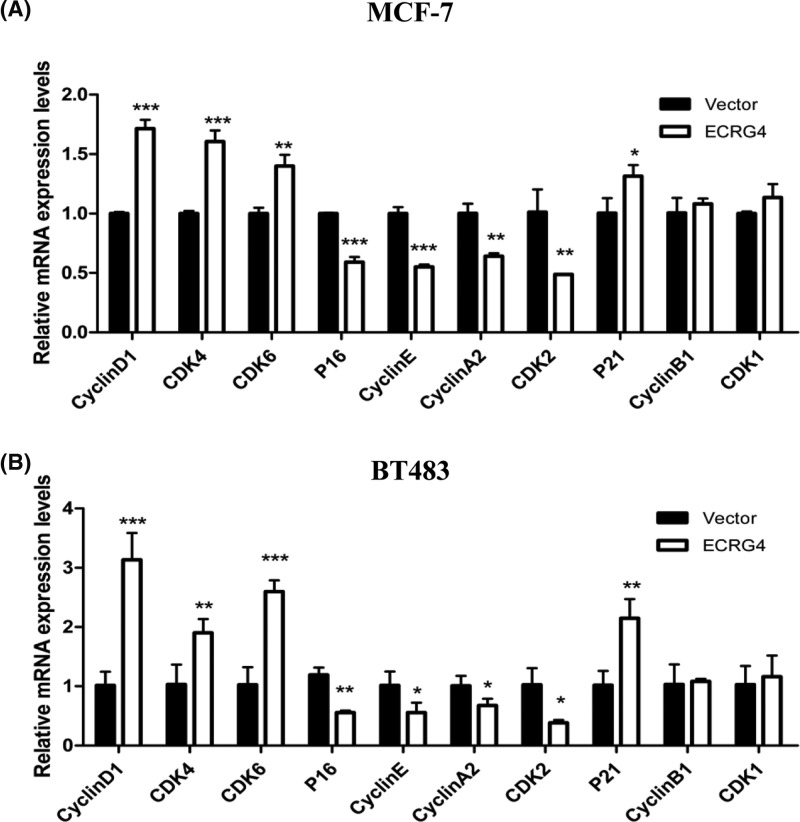
Cell cycle-related gene expression in breast cancer cells (**A**) Expression of cell cycle-related genes in MCF-7 cells as measured by qPCR. (**B**) Expression of cell cycle-related genes in BT483 cells. *P<0.05, **P<0.01, ***P<0.001.

We next analyzed the effect of ECRG4 overexpression on cell cycle progression in breast cancer cell lines by flow cytometry. We found that ECRG4 increased the frequency of G0/G1 phase cells but reduced that of S phase cells. These results suggested that overexpression of ECRG4 may arrest the cell cycle at G0/G1 phase (Figure 8). Subsequently, we assessed the mRNA expression levels of cell cycle-related genes by qPCR (Figure 9). We found that the mRNA levels of CyclinD1, CDK4, CDK6, and p21 in ECRG4-overexpressing cells were elevated while the levels of CyclinA2, CyclinE, P16, and CDK2 were reduced, although levels of CyclinB1 and CDK1 were not significantly altered. These results suggested that overexpression of ECRG4 may lead to a G0/G1 phase block in breast cancer cells.

## Discussion

ECRG4, a gene initially isolated from human esophageal epithelial cells, has been reported as a potential TSG [3–7,11,12]. A meta-analysis by Sabatier et al. [13] reported that ECRG4 expression was decreased in 94.3% of invasive breast cancer. You et al. [14] analyzed 113 tissue samples of primary breast cancers by immunohistochemistry, and found that the loss of ECRG4 expression occurred frequently in tumor tissues (41.6% of tumor samples). Here, we observed that ECRG4 mRNA levels were frequently much lower in tumor tissues (82.4%) than in their normal counterparts.

Recent studies have revealed that silencing of ECRG4 expression in colorectal carcinoma, kidney cancer, and esophageal cancer is associated with the hypermethylation of the ECRG4 promoter region [4–6]. While the specific mechanisms of low expression of ECRG4 in breast cancer samples were unclear, we then explored the association of ECRG4 promoter methylation with ECRG4 expression. We found that the breast cancer samples had a higher frequency of ECRG4 promoter methylation than paired non-tumor tissues, and promoter region methylation status had a negative correlation with ECRG4 mRNA expression level, which was consistent with previous study [15]. Therefore, the hypermethylation of ECRG4 promoter may contribute to the down-regulation of ECRG4 mRNA levels in breast cancer.

To further confirm the link between ECRG4 promoter methylation and expression, MCF-7 and BT-483 cells were chosen and treated by DNA methyltransferase inhibitor 5-Aza-CdR [16–18] and histone deacetylase inhibitor TSA. The MCF-7 and BT-483 cells had low expression level of ECRG4 and exhibited hypermethylation status [19–23]. MCF7 cell line was derived from the pleural effusion of breast adenocarcinoma [20] and BT483 cell line was derived from a primary invasive ductal breast carcinoma [24]. MCF-7 and BT483 cells were estrogen receptor (ER)-positive breast cancer cells [19, 20], and ER positive tumors cells displayed more hypermethylated loci than ER-negative cells [21]. We revealed that each agent alone significantly decreased the methylation of CpG islands in the ECRG4 promoter; thereby, increasing the promoter activity and increasing the expression of ECRG4. Compared with a single drug, these effects were significantly enhanced by combination treatment with 5-Aza-CdR and TSA. These results strongly suggest that reduced ECRG4 expression in breast cancer may be mediated by methylation of the ECRG4 promoter.

We validated that ECRG4 may serve as a potential TSG in breast cancer, and further explored the exact role of ECRG4 in breast cancer cells. We first transfected exogenous ECRG4 into MCF-7 and BT483 cells, and the stably transfected MCF-7 and BT483 cells were identified by fluorescence microscopy and flow cytometry using GFP tag. Overexpression of ECRG4 in MCF-7 and BT483 cells could inhibit their proliferation, which may result from the block at the G0/G1 cell cycle phase, as ECRG4 overexpression led to an enhancement of the frequency of cells in the G0/G1 phase while frequencies of cells in the S phases were reduced. The result was in accordance with the study performed by Li et al. [25]. The cell cycle is rigorously controlled and modulated by cyclins, CDKs, and cyclin-dependent kinase inhibitors (CKI). CDK activity is positively regulated by cyclins and negatively regulated by CKIs [26–28]. Cai et al. revealed that the decreased expression of cyclin D1 and cyclin E may be involved in a G0/G1 phase block induced by ECRG4 in colorectal carcinoma [29].

The G1 phase is the beginning of the cell cycle process, with cyclinD1 being able to combine with CDK4 or CDK6 to promote cell entry into the G1 phase. CyclinE then combines with CDK2 to promote the cell cycle progression into the S phase from the G1 phase [30,31]. CKI p16 can compete with cyclinD1 in combination with CDK4 or CDK6 to inhibit cyclinD1 activation, and p21 can inhibit the activation of cyclin–CDK complexes such as the cyclinE–CDK2 complex in the G1 phase and the cyclinA–CDK2 in the S phase, thus blocking cell cycle progression [30,32,33]. In phase G2, cyclinB1 binding and activating CDK1 further promotes progression into the M phase.

In the context of ECRG4 overexpression, we found that mRNA levels of CyclinD1, CDK4, CDK6, and p21 were increased while levels of CyclinA2, CyclinE, p16, and CDK2 were decreased. We speculate that ECRG4 may suppress the expression of p16 and thereby reduce the inhibitory effect of p16 on cyclinD1–CDK4/6, promoting cell entry into the G1 phase. Moreover, ECRG4 may increase p21 expression to enhance the inhibition of cyclinE–CDK2 and cyclinA–CDK2 in breast cancer. As a result, the cell cycle cannot enter into the S and G2/M phase, leading to arrest in the G0/G1 phase.

Recent studies have validated that ECRG4 induces apoptosis in several cancers through the regulation of Bax and Bcl-2, and through the activation of caspase-3, which cleaves PARP protein [34–36]. Our study shows that ECRG4 promotes apoptosis in breast cancer cell lines. Both an intrinsic mitochondrial pathway as well as an extrinsic death receptor pathway can lead to the induction of apoptosis [37]. The death receptor pathway can be activated through the binding of corresponding death receptors (Fas) and ligands (FasL). Fas can recruit pro-caspase-8 to induce self-shearing resulting in the formation of cleaved caspase-8, which can both activate caspase-3 and Bid; thereby inducing mitochondrial apoptotic pathway activation [38]. In this mitochondrial apoptotic pathway, mitochondrial Cytc is released to form the apoptosome and activate caspase-9. Active caspase-9 further enhances caspase-3-induced PARP cleavage, resulting in cellular apoptosis [39,40]. Bcl-2 can decrease Cytc release to inhibit cell apoptosis through binding with Bax [41]. In our study, we found that PARP was cleaved during ECRG4-induced apoptosis and that overexpression of ECRG4 decreased the expression of the pro forms of caspase 3 and 9 while increasing the levels of cleaved forms of these caspases. In contrast, levels of pro-caspase-8, Fas, FasL, and Bid were not significantly changed in this study. This suggests that the Fas-mediated death receptor pathway is not activated during ECRG4-induced apoptosis. Our results also confirmed that ECRG4 increased Bax expression while suppressing Bcl-2 expression. We additionally observed that Cytc was decreased in the mitochondrial fraction but increased in the cytosolic fraction; whereas, Bax underwent translocation from the cytosol to the mitochondria. We additionally determined that Apaf-1 may interact with caspase-9 and Cytc in these cells, indicating that ECRG4 promoted the formation of the apoptosome. Collectively, our findings indicate that ECRG4-induced apoptosis in breast cancer likely results from the activation of the mitochondrial apoptotic pathway.

Collectively, we found that ECRG4 expression is reduced in human breast cancer samples, possibly as a result of the hypermethylation of the ECRG4 promoter region. We found that overexpression of ECRG4 in MCF-7 and BT483 breast cancer cells inhibit cell growth and migration. Last, we validated that ECRG4 overexpression induces apoptosis, possibly via activation of the mitochondrial apoptotic pathway in these breast cancer cell lines. However, additional research in primary breast cancer samples is needed in order to better understand the link between ECRG4 expression and its promoter methylation status. Moreover, further exploration of ECRG4 methylation profiling as an early diagnostic tumor marker or an indicator of the pathogenesis and prognosis of breast cancer is warranted.

## Conclusion

Our study validated that ECRG4 promoter hypermethylation is a potentially important mechanism governing ECRG4 down-regulation in breast cancer. ECRG4 acts as a TSG in breast cancer, and may induce apoptosis via the mitochondrial apoptotic pathway and block the cell cycle at the G0/G1 phase by regulating the Cyclin–CDK–CKI network. ECRG4 may thus be a pivotal factor in the pathogenesis of breast cancer, and therefore represents a potentially attractive target for therapeutic intervention.

## Supporting information

**supplementary Figure 1:** 

## Abbreviations

5-Aza-CdR: 5-Aza-2′-deoxycytidine
CCK-8: cell counting kit 8
CKI: cyclin-dependent kinase inhibitor
CO-IP: Co-Immunoprecipitation
ECRG4: esophageal cancer-related gene 4
ER: estrogen receptor
GFP: green fluorescent protein
HRP: horseradish peroxidase
MSPCR: methylation-specific PCR
PMSF: phenylmethanesulfonyl fluoride
PVDF: polyvinylidene difluorid
qPCR: quantitative real-time PCR
RT-PCR: reverse transcription polymerase chain reaction
TSA: trichostatin A
TSG: tumor suppressor gene
TBS: tris-buffered saline
