# Supplementary figures and images for: ECRG4 acts as a tumor suppressor gene frequently hypermethylated in human breast cancer
Source: Biosci Rep. 2019 May 10;39(5):BSR20190087. doi: 10.1042/BSR20190087 (PMC6509063; doi:10.1042/BSR20190087)

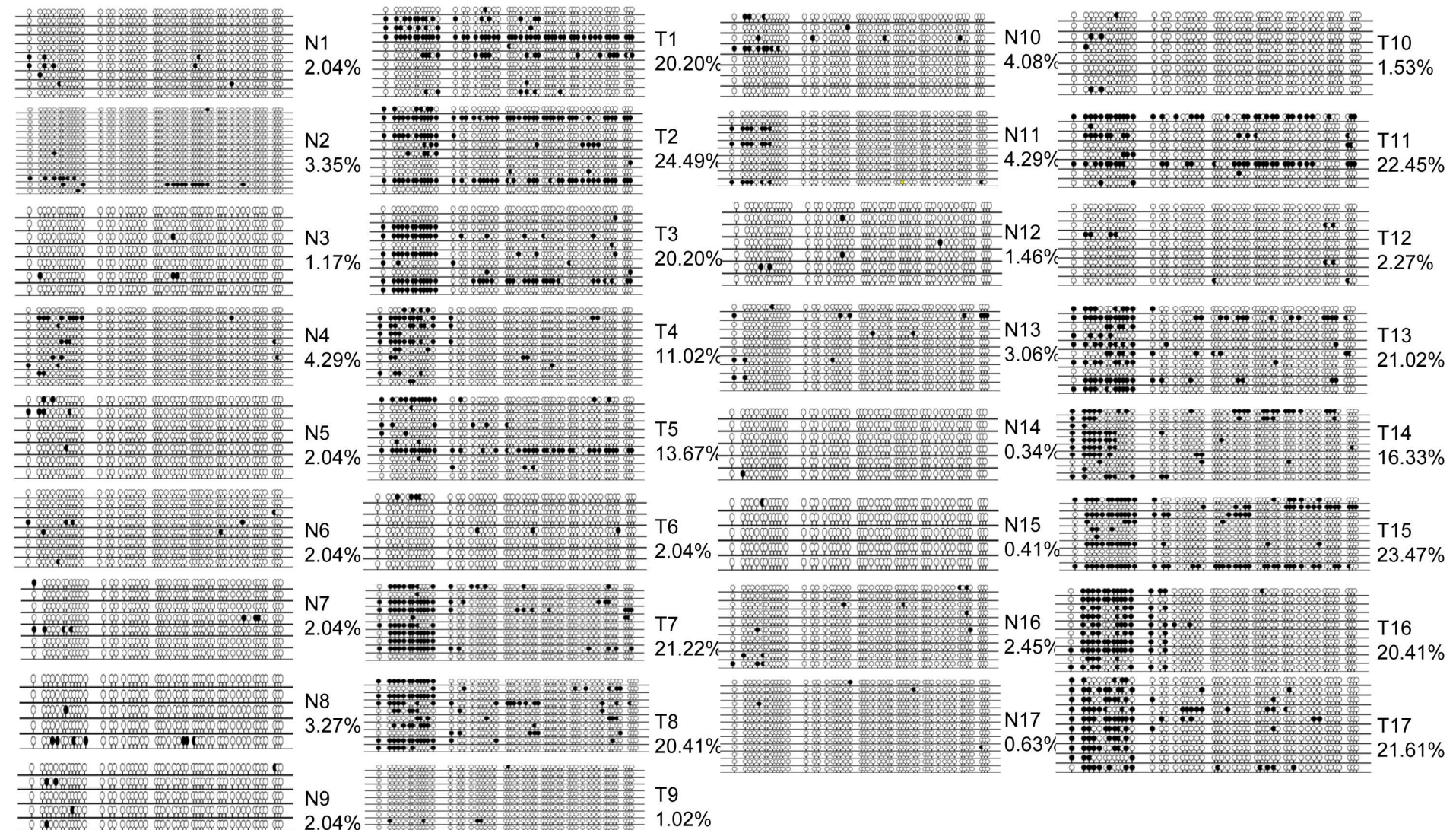

Supplement: Supplementary file 1 [file bsr20190087_Supp1.pdf]
